# CETP Lowers TLR4 Expression Which Attenuates the Inflammatory Response Induced by LPS and Polymicrobial Sepsis

**DOI:** 10.1155/2016/1784014

**Published:** 2016-05-12

**Authors:** Tatiana Martins Venancio, Roberta Marcondes Machado, Angela Castoldi, Mariane Tami Amano, Valeria Sutti Nunes, Eder Carlos Rocha Quintao, Niels Olsen Saraiva Camara, Francisco Garcia Soriano, Patrícia Miralda Cazita

**Affiliations:** ^1^Lipids Laboratory (LIM 10), Faculty of Medical Sciences, The University of São Paulo, São Paulo, SP, Brazil; ^2^Laboratory of Transplantation Immunobiology, Department of Immunology, Institute of Biomedical Science IV, University of São Paulo, São Paulo, SP, Brazil; ^3^Emergency Care Research Unit Laboratory (LIM 51), Faculty of Medical Sciences, The University of São Paulo, São Paulo, SP, Brazil

## Abstract

Sepsis is a systemic inflammatory response to infection eliciting high mortality rate which is a serious health problem. Despite numerous studies seeking for therapeutic alternatives, the mechanisms involved in this disease remain elusive. In this study we evaluated the influence of cholesteryl ester transfer protein (CETP), a glycoprotein that promotes the transfer of lipids between lipoproteins, on the inflammatory response in mice. Human CETP transgenic mice were compared to control mice (wild type, WT) after polymicrobial sepsis induced by cecal ligation and puncture (CLP), aiming at investigating their survival rate and inflammatory profiles. Macrophages from the peritoneal cavity were stimulated with LPS in the presence or absence of recombinant CETP for phenotypic and functional studies. In comparison to WT mice, CETP mice showed higher survival rate, lower IL-6 plasma concentration, and decreased liver toll-like receptor 4 (TLR4) and acyloxyacyl hydrolase (AOAH) protein. Moreover, macrophages from WT mice to which recombinant human CETP was added decreased LPS uptake, TLR4 expression, NF-*κ*B activation and IL-6 secretion. This raises the possibility for new therapeutic tools in sepsis while suggesting that lowering CETP by pharmacological inhibitors should be inconvenient in the context of sepsis and infectious diseases.

## 1. Introduction

Sepsis brings on an uncontrolled inflammatory response and remains a significant cause of death despite the availability of the various therapeutic interventions [1, 2]. The bacterial lipopolysaccharide (LPS) is the primary cause of Gram-negative sepsis, and its interaction with lipopolysaccharide binding protein (LBP) catalyzes the binding of LPS to CD14. The LPS-LBP-CD14 complex presents LPS to toll-like receptor 4 (TLR4) activating an inflammatory gene expression chain through NF-*κ*B and MAPK signaling pathways [3]. Several evidences indicate that the endotoxin LPS is removed from the bloodstream mainly by the liver, and yet the LPS hepatic uptake mechanisms remain uncertain [4–6]. Kupffer cells (KCs) take up most of the free LPS, as well as inactivate LPS by deacylation with acyloxyacyl hydrolase (AOAH) [7]. Plasma LPS is transported in unbound (free) or bound forms. Bound LPS is found in aggregates belonging to bacterial membrane fragments, loosely linked to albumin, CD14, and other proteins. An important fraction of LPS is bound to lipoproteins. Seemingly, the lipoprotein-bound LPS is cleared mainly by hepatocytes [7, 8].

Plasma lipoproteins, especially HDL, are markedly reduced during sepsis. Clinical studies have pointed out that given its anti-inflammatory properties low plasma HDL-cholesterol (HDL-C) is a poor prognostic factor for severe sepsis [8–11]. Infection and inflammation are associated with changes in the activity of plasma proteins that regulate the HDL composition, such as lecithin cholesterol acetyltransferase (LCAT), phospholipid transfer protein (PLTP), and cholesteryl ester transfer protein (CETP) mainly [11–15].

The hydrophobic glycoprotein CETP is bound to HDL in blood and engaged in the transfer of neutral lipids, including cholesteryl ester and triglyceride, amongst lipoprotein particles. Lipoprotein heteroexchange has been implicated in the physiological process of reverse cholesterol transport by which excess of cholesterol removed from peripheral tissues, including the arterial wall, is taken up by the liver and excreted in bile [16]. However, some aspects of the CETP biological function have not been fully understood. High plasma concentrations of CETP are associated with low HDL-C which led to the development of CETP inhibitors that raise HDL-C levels and reduce atherosclerosis in experimental animals [17–20]. However, trials in humans using CETP inhibitors failed to protect against cardiovascular diseases [17, 20–22] and one of them (torcetrapib) increased the incidence of cancer and infection [20, 22]. In addition, recent* in vivo* experiments and human studies have suggested that CETP may have beneficial actions during acute inflammatory states [23, 24]. CETP may play important anti-inflammatory roles for belonging to a protein family that contains other anti-inflammatory proteins, lipopolysaccharide binding protein (LBP), and bactericidal permeability increasing protein (BPI) [24–26]. Accordingly, CETP seems beneficial because in acute inflammatory states mortality rate is decreased after a challenge of LPS to mice expressing human CETP when compared with wild type mice that are known not to express CETP [23].

Considering these facts, in the present study we aimed at clarifying the role of CETP in the inflammatory response and sepsis after cecal ligation and puncture (CLP), a clinically relevant sepsis model. We hypothesize that plasma concentration of CETP influences the TLR4 expression attenuating the inflammatory response induced by LPS and polymicrobial sepsis. For this purpose, we used the human CETP (huCETP) transgenic mouse and a control wild type (WT) focusing our investigation on the role of the innate immune response via TLR4. We anticipated that CETP has a prominent role in the resistance to death induced by CLP by modulating TLR4 activation in the liver.

## 2. Materials and Methods

### 2.1. Experimental Design

Two sets of experiments were designed with sepsis induction by CLP. In a first set of experiments in CLP- or sham-operated mice the survival rates were evaluated. In a second set of experiments, plasma cytokines at 24 h and 48 h after CLP were measured and liver was harvested after exsanguination.

#### 2.1.1. Animal Model

The experimental protocol was approved by the University of São Paulo Medical School Ethics Committee (029/12). Hemizygous human CETP transgenic mice (line 5203, C57BL6/J background) [27] expressing a human CETP minigene under the control of natural flanking sequences were derived from Dr. Alan R. Tall's colony (Columbia University, New York, NY) and crossbred with wild type mice C57BL6/J from the Experimental Animal Center of Faculty Medical Science of University of São Paulo. The mouse tail blood was also drawn for the determination of the plasma CETP concentration and activity [28]. Male littermates, 8–12 weeks of age, expressing human CETP (+) and wild type (WT) control mice that are known for not expressing CETP were housed in a temperature-controlled room under a 12-h light-dark cycle with free access to a standard chow diet (Nuvital, PR, Brazil) and water.

#### 2.1.2. CETP Activity Assay and Concentration

Plasma CETP activity measured by an exogenous method mirrors the plasma CETP concentration [29]. Briefly, a mixture of human very low density lipoprotein (VLDL) and low density lipoprotein (LDL) in 200 *µ*L (200 mg/dL cholesterol) was incubated with pooled HDL particles from human donors (50 *µ*L: 40 mg/dL cholesterol) previously labeled with [4-^14^C]-CE, mouse plasma (10 *µ*L) as the source of CETP, and Tris buffer (40 *µ*L) in a final volume of 300 *µ*L. Blanks were prepared with Tris/saline/EDTA buffer (10/140/1 mM), pH 7.4, and control plasma from wild type C57BL/6 mice that does not express CETP. Incubations were carried out at 37°C for 2 h. The apoB-containing LP was precipitated with a 1.6% dextran sulfate/1 M MgCl_2_ solution (1 : 1) and radioactivity measured in the remaining supernatant in a scintillation solution (Ultima Gold, Eastman Kodak Co., Rochester, NY) in the LS6000 Beckman Beta Counter (Beckman Instruments, Palo Alto, Calif). The percentage of [4-^14^C]-CE transferred from [^14^C]-CE-HDL to VLDL + LDL was calculated as [1 − (sample radioactivity/control radioactivity) × 100].

CETP concentration (mass) was measured by a commercially available enzyme-linked immunosorbent assay kit (Wako Chemicals USA, Inc.) according to the manufacturer's protocols.

Animals used in this study were selected according to their plasma CETP mass and activity. In CETP+ versus WT (CETP-) mice, respectively, the CETP concentration (*µ*g/mL ± SD) was 5.80 ± 2.07 versus 0.025 ± 0.023 and percent activity was 42.00 ± 14.54 versus 8.42 ± 4.03 by Mann-Whitneytest (*p* < 0.01). The plasma CETP activity was positively related to CETP concentration (*Pearson r. *0.9527,* p* < 0.001) confirming that plasma CETP activity mirrors the plasma CETP concentration [23, 28, 29]. These mice show a moderate decrease in HDL-C and elevation in apoB-containing lipoprotein [28].

#### 2.1.3. Induction of Sepsis and Survival Rate Assay

To investigate the role of the expression of human CETP in the host defense* in vivo*, mice huCETP and WT, matched for gender and age (male, 8 to 12 weeks old), were submitted to experimental sepsis by cecal ligation and puncture (CLP) [30]. Briefly, mice were anesthetized using ketamine (60 mg/kg, i.p., Ketalar, Parke-Davis, São Paulo, Brazil) and xylazine (10 mg/kg, Rompum, Bayer S.A., São Paulo, Brazil) and the caecum was exposed, ligated 1 cm above its extremity, and punctured once with a 24-gauge needle, characterizing a midgrade mortality procedure [30], and the incision was closed with surgical staples. The controls of CLP were sham-operated. Sham mice underwent abdominal incision and caecum exposure without ligation and puncture. After the procedure, mice had access to water and food* ad libitum*. Survival was monitored for five days every 8 hours, while, for cytokines and organ harvesting, a different group of animals were anesthetized, bled, and sacrificed after CLP (*n* = 6–8) at 24 or 48 h depending on the time point analyzed.

#### 2.1.4. Measurements of Cytokines and LBP

The levels of LBP and cytokine IL-6 in plasma and cell supernatants were measured by standard sandwich ELISA kit commercially available (R&D Systems, Minneapolis, MN) according to the manufacturer's protocol.

#### 2.1.5. Determination of Protein Expression of TLR4 and AOAH by Western Blot Analysis

After CLP, the livers from animals were removed, stored in liquid nitrogen, and fragmented in tissue spray (Mikro-Dismembrenator II, B. Braun Melsungen, FRG), after adding Tris-buffered saline plus 1x inhibitors protease (1.5 *µ*M aprotinin, 1 *µ*M leupeptin, 2 *µ*M pepstatin and 0.1 mM phenylmethanesulfonyl fluoride (PMSF)), centrifuged, and diluted in SDS glycerol. Samples were maintained at −70°C until processing to quantify the total protein concentration by BCA method solution standard. Equal amounts of hepatic cellular protein (40 *µ*g) were applied to 10% polyacrylamide gel (SDS-PAGE) and separated by electrophoresis (150 V, 1 hour).

The expressions of TLR4, AOAH, and *β*-actin were determined using primary antibodies anti-TLR4 and AOAH (Abcam, Cambridge, MA, EUA), as well as anti-*β*-actin (Fitzgerald Industries International Inc., Concord, MA) at 1 : 1000 dilution. The respective secondary antibodies were used at 1 : 5000 dilution (GE Healthcare, Uppsala, Sweden). Proteins were detected with horseradish peroxidase-conjugated antibodies and an enhanced chemiluminescence reagent (Pierce, Rockford, IL). Protein density was quantified with an ImageQuant 300 Imager (*GE Healthcare*) and differences between bands were analyzed in pixels, utilizing the ImageQuant TL Software (*GE Healthcare*). Results are expressed as arbitrary units and normalized for *β*-actin expression.

#### 2.1.6. Cell Culture

Peritoneal macrophages isolated from male C57BL6/j mice (8–12-week-old) were harvested after the injection of 6 mL of PBS into the peritoneal cavity. After a soft abdominal massage, cells were collected into sterile tubes, centrifuged, and diluted in RPMI 1640 containing 10% fetal calf serum (FCS), 1% penicillin-streptomycin, and 4 mM L-glutamine. The cells were then plated in 48-well plates and maintained in a 5% CO_2_ incubator at 37°C. During the different experiments reported below, cells were maintained in RPMI and cell viability was assured by lactate dehydrogenase release to cell culture medium (*In Vivo* Toxicology Assay Kit, Sigma Aldrich, St. Louis, MO, USA) in all experimental conditions. No change in apoptosis was observed after exposure to LPS and recombinant CETP (data not shown).

#### 2.1.7. Confocal Microscopy

CETP belongs to a protein family that contains two other anti-inflammatory proteins, lipopolysaccharide binding protein (LBP) and bactericidal permeability increasing protein (BPI) [25]. Confocal microscopy was assessed due to the possibility of interaction between CETP and LPS in the macrophages. Peritoneal macrophages from WT mice were stimulated with LPS (1 *µ*g/mL) in the presence of recombinant LBP which was utilized as a positive control (Cell Sciences Inc., USA) or recombinant human CETP (Roar Biomedical, Inc., USA): 1 *µ*g/mL diluted in RPMI at 37°C for 4 hours. The cells were washed twice with cold PBS and fixed in 200 *µ*L of 4% paraformaldehyde in 100 mM sodium phosphate, pH 7.4, for 30 min at room temperature and were next blocked with 1x PBS containing 1% BSA, 0.2% Triton, for 30 min at room temperature. The cells were incubated with primary antibodies overnight at 4°C: CETP: mouse polyclonal anti-CETP human (Abnova, Walnut, CA, USA), LBP: Polyclonal Goat anti-mouse (R&D Systems, USA), and Macrophage: rat anti-mouse Alexa Fluor 647-conjugated (Serotec, USA) were used at 1 : 200; 1 : 100, and 1 : 400 dilutions, respectively. Next, cells were washed 3 times with PBS, labeled with the respective secondary antibodies: Alexa Fluor 350-conjugated rabbit anti-mouse (Invitrogen, Eugene Oregon, USA) and Cy3-conjugated donkey anti-goat (Abcam, Cambridge, MA, USA.). Nuclei were counterstained with 4′,6′-diamino-2-phenylindole (DAPI, 1 *μ*g/mL in 1x PBS) for 20 minutes at room temperature. The slides were prepared with one drop of the glycerol solution (9 vol of glycerol, 1 vol of 1 M Tris-Cl) in microscope cover glasses (Fisher Scientific, Pittsburgh, PA) kept in the dark until further analysis. Confocal microscopy was then carried out using laser confocal imaging system (Zeiss, LSM 510 Meta Laser Scanning, Germany), magnification of 600x.

#### 2.1.8. Flow Cytometry

Peritoneal macrophages isolated from male C57BL6/j mice (8-week-old) were treated with 1 *µ*g of LPS (Alexa Fluor 488,* E. coli* Serotype O55:B5, Invitrogen Molecular Probes, Eugene, Oregon, USA) in the absence or presence of various concentrations of recombinant human CETP (*Roar Biomedical, NY, USA*) (0.1, 0.5, and 1.0 *µ*g/mL) diluted in RPMI and stained with the monoclonal antibodies; we utilized anti-mouse F4/80 PerCP/Cy5.5 a monoclonal antibody directed specifically against the mouse macrophage; for TLR4 we utilized anti-TLR4-PE (BD Biosciences, Franklin Lakes, NJ, USA). NF-*κ*B: rabbit anti-mouse Phospho-NF-*κ*B p65 Alexa Fluor 647 Conjugate (Cell Signaling Technology, Inc., Boston, MA, USA) was diluted at 1 : 100. We utilized Cytofix (*BD Biosciences, San Jose, USA*) for surface label and Cytofix/Cytoperm (*BD Biosciences, San Jose, USA*) for intracellular label. Basal fluorescence was determined in unmarked cells and compensation made with cells labeled with the respective fluorochromes. Samples were acquired on FACSCanto, using FACSDiva software (BD Biosciences) and then were analyzed with FlowJo software (Tree Star, Ashland, OR, USA). Fluorescence voltages were determined using matched unstained cells. Fifty thousand events were acquired in a live mononuclear gate.

#### 2.1.9. Statistical Analysis

Statistical analyses were performed using the GraphPad software version 5.0 (GraphPad Software Inc., San Diego, CA, USA). Results are presented as mean ± SD. Data were analyzed using a one-way ANOVA or Student's* t-*test. Differences were considered statistically significant with values of *p* < 0.05. Animal survival was analyzed by Kaplan-Meier survival analysis and log-rank test.

## 3. Results

### 3.1. *In Vivo* Study

#### 3.1.1. Human CETP (huCETP) Expressing Mice Are More Resistant Than Wild Type Mice to Polymicrobial Sepsis

**Figure 1:**
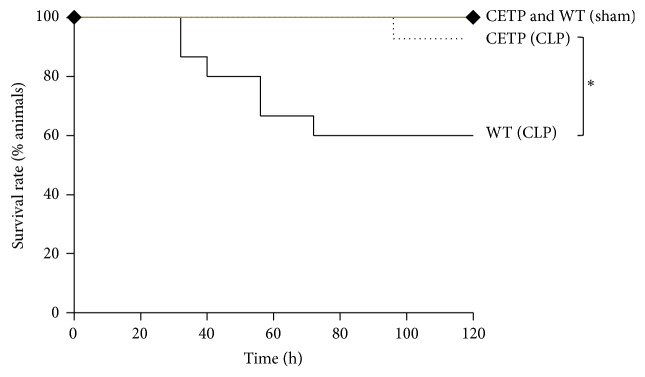
Human CETP expressing mice are more resistant to polymicrobial sepsis than wild type mice. CETP and WT mice submitted to CLP and control mice were sham-operated on (*n* = 8). Survival rate study of mice monitored every 8 hours for 5 days after CLP. ^*∗*^ *p* = 0.0267 by a log-rank test between CLP groups. Mortality rate in the sham group did not change.

To investigate the CETP relevance on sepsis, mice expressing the human CETP (huCETP) transgene and wild type (WT) mice were submitted to CLP and to sham-operation utilized as control for both groups. Along 5 days the survival rate of CETP mice was significantly higher than that of WT mice, 93.3 versus 60.0 (%), respectively. No difference was observed in the survival rate in the control groups (sham-operated). Human CETP expressing mice were more resistant to the CLP induced lethality, which is compatible with a beneficial role of the human CETP expression (Figure 1).

**Figure 2:**
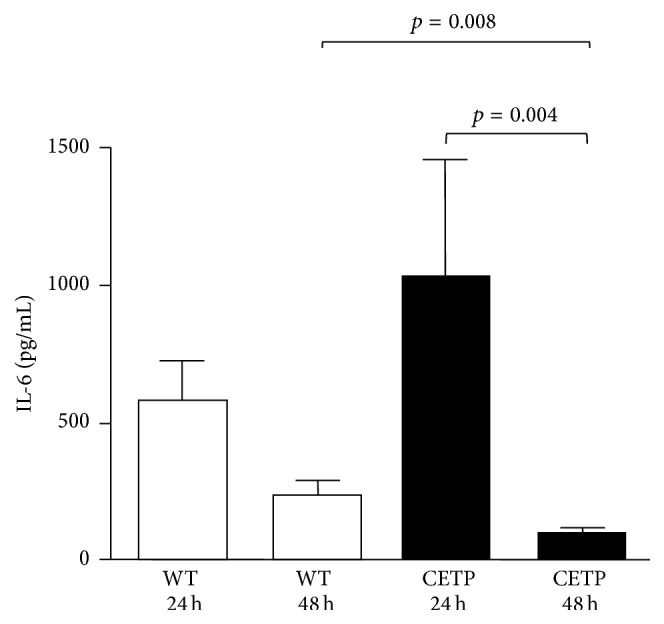
Plasma IL-6 levels in CETP and WT mice after CLP induced polymicrobial sepsis. Plasma IL-6 levels were measured at 24 h and 48 h after CLP by ELISA. Results are expressed as means ± SD, *n* = 6 mice per group. Data were analyzed by one-way ANOVA (*p* = 0.008: WT 48 h versus CETP 48 h; *p* = 0.004: CETP 24 h versus CETP 48 h).

To further evaluate whether the CETP expression modulates* in vivo* the immunoinflammatory response to CLP, proinflammatory cytokine IL-6 was measured; blood was then drawn at indicated times from CETP and WT mice. IL-6 plasma concentrations at 48 h were lower in animals that express CETP than in WT mice and also in those expressing CETP were lower at 48 h than at 24 h (Figure 2).

**Figure 3:**
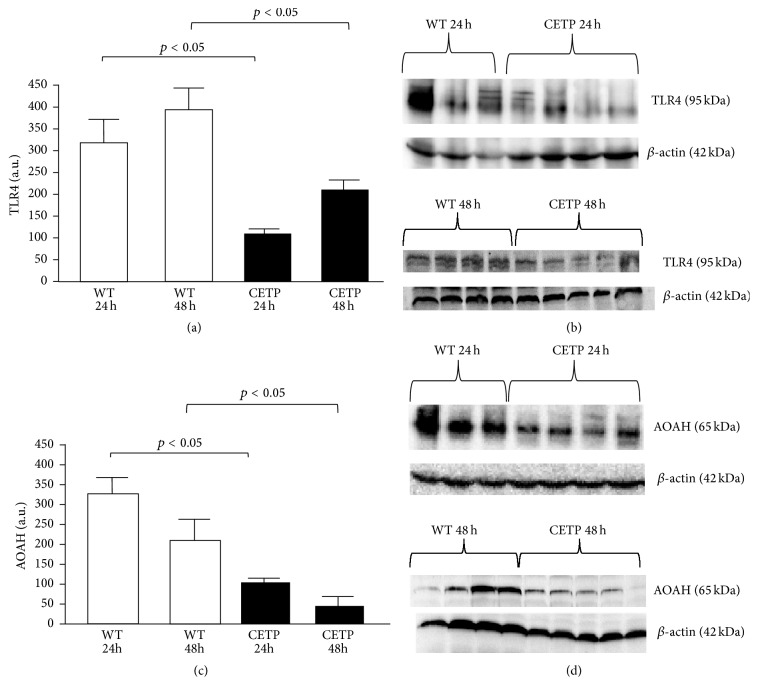
The hepatic content of TLR4 and AOAH decreased in the liver from CETP as compared to WT mice. Liver samples were harvested at 24 h and 48 h after CLP. TLR4 ((a) and (b)) as well as AOAH ((c) and (d)) protein expression was determined by Western blotting. Graph presented as mean ± SD of arbitrary units corrected by *β*-actin level ((a) and (b)); *n* = 3–5, differences by one-way ANOVA, and *p* < 0.05. ((b) and (d)) Representative Western blot images of TLR4, AOAH, and *β*-actin protein expression.

The hepatic content of* TLR4* and of* AOAH* markedly decreased in the liver from CETP as compared to WT mice. Inflammation induced by CLP in a mouse model activates toll-like receptor 4 (TLR4) in the liver leading to progression of acute liver failure and worsening of sepsis. There is evidence that bacterial LPS (endotoxin) is removed from the bloodstream mainly by the liver and that the LPS-inactivating enzyme (acyloxyacyl hydrolase, AOAH) is mainly produced by the liver Kupffer cells (KCs) [7]. Thus, after CLP induced polymicrobial sepsis at 24 h and 48 h, the liver protein levels of TLR4 and of AOAH were determined and shown markedly decreased in CETP as compared to WT (Figure 3).

### 3.2. *In Vitro* Study

As the* in vivo* experiments revealed an influence of CETP on endotoxemia and CLP induced sepsis, we asked whether this would have an impact on cell activation in the TLR4 signaling cascade. To explore the mechanism by which the CETP expression prolongs the survival rate of the transgenic mice, we analyzed the role of CETP in the inflammatory response in mouse peritoneal macrophages stimulated with LPS and treated with recombinant human CETP upon measuring cell LPS uptake, TLR4 expression, NF-*κ*B activation by flow cytometry, and release of cytokines in LPS-stimulated macrophages.

#### 3.2.1. Confocal Microscopy Colocalization of LPS and CETP in Macrophages

**Figure 4:**
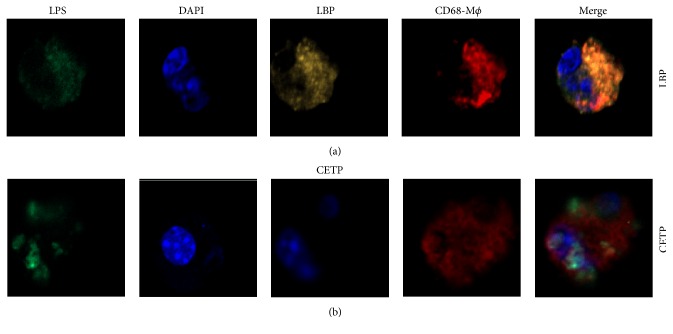
Confocal microscopy of macrophages: colocalization between LPS and CETP (magnification of 600x). Representative confocal image of peritoneal macrophages from WT mice stimulated with LPS (1 *µ*g/mL) for 4 h in the presence of the LBP, known to be attached to LPS: LBP was utilized as a positive control (a) or CETP (b) (1 *µ*g/mL). FITC-LPS, green; nucleus, blue (DAPI); LBP, yellow; CD68^+^ macrophages, red; CETP, blue (in the absence of DAPI because CETP antibody is also blue); merge: colocalization between markers.

We assessed the possible interaction between CETP and LPS in the macrophages. LBP (yellow), known to be attached to LPS, utilized as a positive control, is shown along the marked regions on the plasma membrane with colocalization by CD68 (red) and LPS (green) (Figure 4(a)). CETP (blue in the absence of DAPI) appears in the perinuclear region and has a partial colocalization with LPS (green) (Figure 4(b)) by a confocal microscopy. We demonstrate for the first time that CETP interacts with LPS in macrophages.

The plasma levels of LBP were measured by ELISA in 6 animals in each group. Basal levels of plasma LBP (*µ*g/mL ± SD) from CETP and WT mice did not differ: 31.58 ± 18.08 and 12.65 ± 5.07, respectively. After CLP, we observed markedly increased plasma LBP concentration in CETP and in WT mice as compared to the sham mice: 295.6 ± 8.6 versus 84.99 ± 6.11 (CETP) and 291.3± 28.32 versus 80.27 ± 22.58 (WT) (differences by Mann-Whitney test; *p* < 0.01). However, LBP did not differ between CETP expressing and WT mice (data not shown).

#### 3.2.2. Effects of the Presence of CETP on the LPS Uptake, TLR4 Expression, NF-*κ*B Activation, and Release of Cytokines in LPS-Stimulated Macrophages

**Figure 5:**
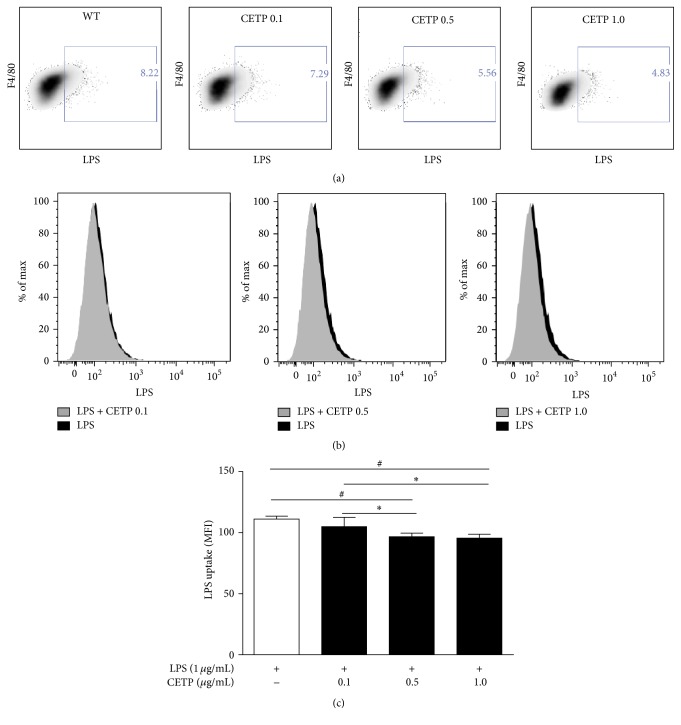
CETP reduces macrophage LPS uptake after LPS stimulus. Peritoneal macrophages obtained from WT mice were stimulated for 24 h with LPS-Alexa Fluor® 488 (1 *µ*g/mL) in the absence or presence of CETP (0.1, 0.5, and 1.0 *µ*g/mL). (a) Representative FACS dot plots gated on F4/80^+^ cells (macrophages only) and the percentage of LPS-Alexa 488^+^ cells are shown. (b) Representative flow cytometry histograms comparing macrophages (F4/80^+^) from WT and respective CETP groups stimulated with LPS. (c) Graph presented as mean ± SD of MFI (median fluorescence intensity) analyzed using FACSDiva software (*n* = 4) representative of three independent experiments, by one-way ANOVA *p* = 0.0014 and posttest (Newman-Keuls Multiple Comparison Test; ^*∗*^ *p* < 0.05 and ^#^ *p* < 0.01).

**Figure 6 fig6:**
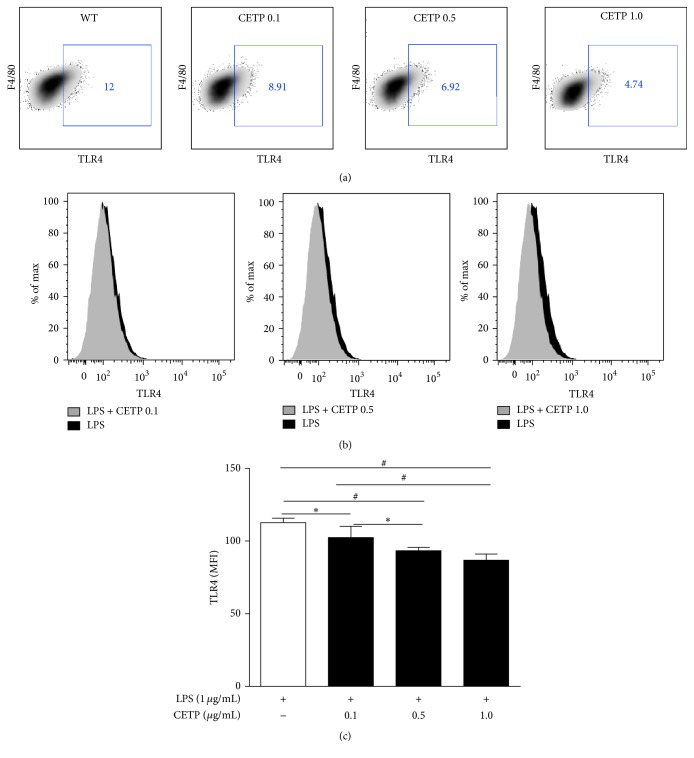
CETP reduces TLR4 expression in LPS-stimulated macrophages. Peritoneal macrophages obtained from WT mice were stimulated for 24 h with LPS-Alexa Fluor 488 (1 *µ*g/mL) in the absence or presence of CETP (0.1, 0.5, and 1.0 *µ*g/mL). (a) Representative FACS dot plots gated on F4/80^+^ cells (macrophages only) and the percentage of TLR4^+^ cells are shown. (b) Representative flow cytometry histograms of TLR4 comparing macrophages (F4/80^+^) from WT and respective CETP groups stimulated with LPS. (c) Graph presented as mean ± SD of MFI (median fluorescence intensity) analyzed using FACSDiva software (*n* = 4), representative of three independent experiments, by one-way ANOVA *p* = 0.0014 and posttest (Newman-Keuls Multiple Comparison Test; ^*∗*^ *p* < 0.05 and ^#^ *p* < 0.01).

**Figure 7 fig7:**
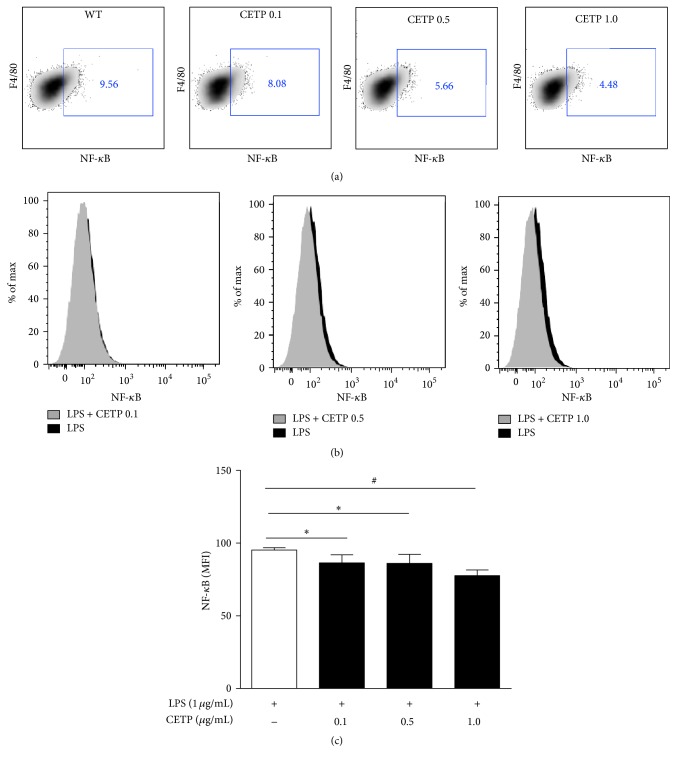
CETP reduces LPS-stimulated transcription factor NF-*κ*B activation. Peritoneal macrophages obtained from WT mice were stimulated for 24 h with LPS-Alexa Fluor 488 (1 *µ*g/mL) in the absence or presence of CETP (0.1, 0.5, and 1.0 *µ*g/mL). (a) Representative FACS dot plots gated on F4/80^+^ cells (macrophages only) and the percentage of* NF-κB* ^+^cells are shown. (b) Representative flow cytometry histograms of* NF-κB* comparing macrophages (F4/80^+^) from WT and respective CETP groups stimulated with LPS. (c) Graph presented as mean ± SD of MFI (median fluorescence intensity) analyzed using FACSDiva software (*n* = 4) representative of three independent experiments, by one-way ANOVA *p* = 0.0019 and posttest (Newman-Keuls Multiple Comparison Test; ^*∗*^ *p* < 0.05 and ^#^ *p* < 0.01).

**Figure 8:**
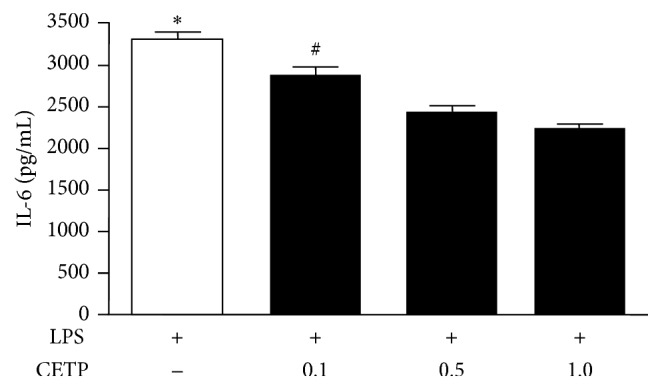
CETP reduces the release of IL-6 levels in LPS-stimulated macrophages. Peritoneal macrophages from WT mice were stimulated for 24 h with LPS-Alexa Fluor 488 (1 *µ*g/mL) in the absence or in the presence of different concentrations of recombinant human CETP (0.1, 0.5, and 1.0 *µ*g/mL). IL-6 concentration release to cell culture medium decreased dose dependently in the presence of CETP (*n* = 4). Graph presented as mean ± SD representative of three independent experiments. Data were analyzed by one-way ANOVA *p* = 0.0014 and posttest (Newman-Keuls Multiple Comparison Test; ^*∗*^ *p* < 0.05: control LPS versus CETP group; and ^#^ *p* < 0.01: CETP 0.1 versus CETP 1.0).

It is known that the LPS/TLR4 complex plays a vital role in initiating LPS signaling during inflammation bringing about NF-*κ*B activation. The phosphorylation of the p65 subunit is critical for cytoplasmic migration to nuclei thus initiating transcription of downstream target genes. Further, we examined LPS uptake, TLR4 expression, activation of the NF-*κ*B, and release of cytokines in LPS-stimulated macrophages in the absence or in the presence of CETP. Flow cytometry revealed that LPS uptake (Figure 5), expression of TLR4 (Figure 6), NF-*κ*B activation (Figure 7), and release of IL-6 (Figure 8) in the presence of different concentrations of CETP (0.5 and 1.0 *µ*g/mL) were attenuated indicating that CETP interferes with the LPS uptake and the TLR4 signaling cascade (Figures 5 [Fig fig6] [Fig fig7]–8).

## 4. Discussion

In spite of several studies, the interaction of the lipoprotein with risk factors in sepsis is not clear. In sepsis there is a reduction in plasma concentrations of lipoproteins, especially HDL-C, which is due in part to alterations in the activity of plasma proteins that modulate the metabolism of these lipoproteins, such as CETP [19]. CETP has structural homology with LBP that takes part in the innate immune response by binding to LPS, triggering the inflammatory response mediated by TLR4 and culminating in the activation of the transcription factor NF-*κ*B [31].

A previous study from our laboratory showed that after intraperitoneal injection of LPS human CETP expressing mice were more resistant to inflammation compared to control mice [23] indicating that CETP probably has functions other than those related to plasma lipoprotein metabolism as suggested by other authors [32]. Also, Grion and colleagues [24] showed that, in patients with sepsis, CETP is decreased in nonsurvivors as compared to survivors.

In the present study, mice transgenic for human CETP (huCETP) and nontransgenic sibling controls (WT) were subjected to CLP, and the survival rate and the inflammatory profiles were assessed. The CLP model used has characteristics similar to those of the human peritonitis [30]. CETP mice showed greater resistance to CLP induced sepsis, with a survival rate of 93.3% versus 60% of the WT group, confirming previous findings in the experimental model of endotoxemia [23]. Thus, we investigated the inflammatory profile to understand how CETP is involved in sepsis protection. Accordingly, confirming the findings of the endotoxemia study [23], we observed a sharp drop in the concentration of plasma IL-6 in CETP animals (*p* = 0.004) at 48 hours after surgery, which was not observed in WT group.

The sharp decrement of the IL-6 value in huCETP mice at 48 hours contributes to explaining the resistance to death in this group whereas in the WT mice the death rate increased shortly before the 40th hour and markedly raised thereafter (Figure 1). The behavior of IL-6 in the inflammatory response is variable along time as shown by Fontes et al. [33], according to whom “chronically elevated IL6 levels lead to chronic inflammation and fibrotic disorders. Thus, IL6 can be both protective and pathogenic, depending on the kinetics of the host response.”

IL-6 is directly related to the risk of death in patients with sepsis and IL-6 blockade protected against this disease [34], suggesting that huCETP animals are resistant to sepsis induced death due to lower plasma concentrations of IL-6 in 48 hours. Furthermore, differences between* in vitro* and* in vivo* studies could be ascribed to the medium utilized for IL-6 measurements: plasma was utilized in experiments* in vivo* whereas cell cultured medium was utilized* in vitro*. The LPS stimulus brings about a faster inflammatory response* in vitro* when compared to the* in vivo* response to CLP stimulus. More IL-6 is produced by the LPS stimulus* in vitro* either in the presence or in the absence of CETP. However, production of IL-6 diminishes in the presence of recombinant CETP dose dependently (Figure 8). Furthermore, there is the possibility that IL-6 is associated with the production of iNOS due to the fact CETP anti-inflammatory action reduces NF-*κ*B which in turn would modify iNOS [35]. This possibility needs to be explored in future research.

There are several evidences supporting that LPS is removed from the bloodstream mostly by the liver, although the mechanisms remain uncertain. Our previous study [36] showed that the plasma clearance of ^3^H-LPS was faster at 24 hours in CETP animals being taken up predominantly in the liver. In that study, it was observed that LPS was transported by HDL mainly and also by LDL particles but little by VLDL particles a fact that may have contributed to minimizing the adverse effects of LPS. Alves-Filho et al. [37] demonstrated the detrimental role of TLR4 in the development of infection by polymicrobial sepsis because TLR4-mediated signaling impaired the migration of neutrophils to the infection site. Moreover, reduced expression of TLR4 in the liver improves the regeneration of this organ in rats subjected to CLP induced sepsis [38]. We have also shown that in the liver huCETP mice showed reduction of TLR4 as compared to WT mice. Although our previous study [36] had demonstrated increased clearance and hepatic uptake of radioactive LPS in mice expressing huCETP, we can speculate that in the presence of CETP somehow LPS is processed by the liver cells and secreted into the bile [5] reducing the activation of LPS in Kupffer cells [7]. CETP possibly prevents prolonged inflammatory response by mechanism that not only involves the ability of AOAH in deacylating LPS but moves LPS to the liver through another pathway responsible for its detoxification.

In the present experimental study, there was a surge in the plasma IL-6 level after CLP and a simultaneous increase of liver TLR4 which was not hampered by an increase in liver AOAH [34, 35, 39] thus indicating that TLR4 in modulating hepatic inflammation is harmful to WT mice and is diminished in CETP animals.

**Figure 9:**
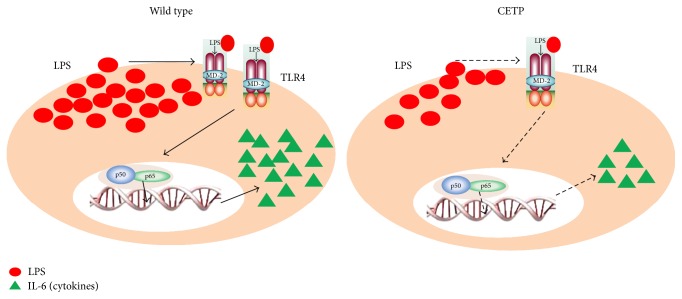
The presence of CETP reduced the cellular uptake of LPS, TLR4 expression, and NF-*κ*B activation (p65) and consequently decreased the secretion of IL-6 indicating that CETP interferes in TLR4 signaling pathways.

Given these findings* in vivo*, we sought to clarify* in vitro* the involvement of CETP in the inflammatory response triggered by LPS and mediated by TLR4. We show here that CETP is able to interact with LPS in macrophages (Figure 4). According to flow cytometry, it was observed that macrophages stimulated with LPS and added recombinant CETP reduced the cellular uptake of LPS, TLR4 expression, and NF-*κ*B activation and consequently decreased the secretion of IL-6 into the cell culture medium (Figure 9) [40]. These data strongly suggest that CETP in macrophages as well as in liver (found* in vivo*) prevents the interaction of LPS with TLR4, thereby reducing the inflammatory response.

## 5. Conclusion

Considering the limitations to the human physiology of our observations in mouse models, we present here original data indicating that CETP shields plasma LPS from triggering septic responses, therefore contributing to explaining, in part, the unfavorable results of the pharmacological inhibition of CETP in humans.

## Abbreviations

CETP: Cholesteryl ester transfer protein
LP: Lipoprotein
HDL: High density lipoprotein
TLR4: Toll-like receptor 4
CLP: Cecal ligation puncture.
